# Coherent Terahertz Detection via Ultrafast Dynamics of Hot Dirac Fermions in Graphene

**DOI:** 10.1021/acsnano.3c08731

**Published:** 2024-02-01

**Authors:** Mark D. Thomson, Florian Ludwig, Jakob Holstein, Reiam Al-Mudhafar, Shihab Al-Daffaie, Hartmut G. Roskos

**Affiliations:** † Physikalisches Institut, Johann Wolfgang Goethe-Universität, 60438 Frankfurt am Main, Germany; ‡ Department of Electrical Engineering and Center for Terahertz Science and Technology, Eindhoven University of Technology, 5612 AE Eindhoven, Netherlands

**Keywords:** graphene, terahertz, optoelectronic, coherent detection, photoconductive antenna

## Abstract

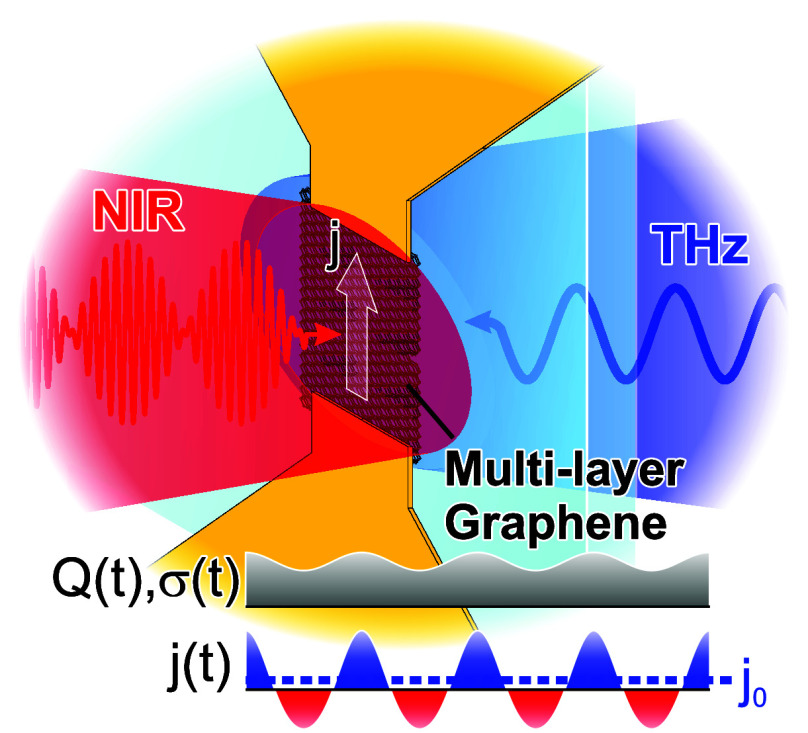

Graphene has recently been shown to exhibit ultrafast conductivity modulation due to periodic carrier heating by either terahertz (THz) waves, leading to self-induced harmonic generation, or the intensity beat note of two-color optical radiation. We exploit the latter to realize an optoelectronic photomixer for coherent, continuous-wave THz detection, based on a photoconductive antenna with multilayer CVD-grown graphene in the gap. While for biased THz emitters the dark current would pose a serious detriment for performance, we show that this is not the case for bias-free THz detection and demonstrate detection up to frequencies of at least 700 GHz at room temperature, even without optimized tuning of the doping. We account for the photocurrent and photomixing response using detailed simulations of the time-dependent carrier distribution, which also indicate significant potential for enhancement of the sensitivity, to become competitive with well-established semiconductor photomixers.

Graphene possesses two key aspects for ultrafast optoelectronic applications. First, one can obtain a drastic change in the carrier temperature, *T*_el_, and concomitant electronic conductivity, with only modest incident radiation intensities,^1,2^ applicable over a huge spectral range due to the (near-)gapless band structure. Second, the cooling of the carriers due to energy transfer to the lattice (which has a much higher heat capacity) can take place on a time scale τ_c_ ≲ 1 ps,^3−5^ allowing for response bandwidths reaching the THz range.^6,7^ In addition to realizing conventional fast power-law photodetectors for the infrared (IR)/visible range,^7,8^ the ultrafast conductivity modulation can interact with incident GHz/THz-waves to manifest as a strong nonlinear wave-mixing effect. This was demonstrated in single-layer graphene with high-field THz pulses,^9^ derived from an accelerator-based undulator source with center frequencies in the range 0.3–0.68 THz. In this case, the THz waves both drive and subsequently mix with the modulation to manifest as harmonic generation, with odd harmonic orders up to *n* = 7 being observed. The nonlinear response was found to be dependent on the doping (Fermi level) of the charge carriers,^10^ and could be described by a model accounting for the time-dependent conductivity of the hot-carrier distribution and the resulting current density.^11^ Due to the high 2D carrier density in a graphene monolayer, the effective bulk nonlinear susceptibilities χ^(*n*)^ are very high, although this is not specifically due to the Dirac band structure, as one arrives at similar magnitudes of χ^(*n*)^/*N* per carrier in conventional bulk semiconductors with parabolic bands (as demonstrated recently in weakly p-doped Si^12^).

Besides such THz self-mixing, the conductivity modulation can be driven by superposing the light from two continuous-wave (CW) optical lasers (at frequencies ν_1,2_) to obtain a beat note at the difference-frequency ν = ν_2_ – ν_1_. Indeed, this was demonstrated to achieve a strong periodic modulation of *T*_el_,^5,13^ and while at low temperature τ_c_ > 10 ps, at room temperature it is much faster i.e., τ_c_ ∼ 1 ps. Here, we explore the potential to realize the coherent detection of CW THz waves, as depicted in Figure 1a, by photomixing the optically driven conductivity modulation from two near-IR lasers with CW THz waves at the same frequency. The conductivity in the photomixer gap is given (to first order) by σ(*t*) = σ_0_ + Δσ cos(ω*t* + φ) (φ being the phase offset between the conductivity modulation and incident THz wave, ω = 2πν), where the photoconductivity Δσ can be either positive or negative depending on the initial doping (Fermi level),^1,4^ as discussed in detail below. With an additional incident THz field, *E*_THz_ (assumed to drive carriers at the Fermi surface classically via the Coulomb force), one then obtains an oscillatory current density *j*(*t*) = σ(*t*)*E*_THz_(*t*), which contains *even* harmonics *j*_2*n*_, where the rectified component *j*_0_ ∝ Δσ·*E*_0_ cos(φ) (coherent and linear in the THz field amplitude *E*_0_) can be measured in the external circuit, i.e., the optical beat note demodulates the THz field to base band frequencies as per conventional THz photomixers.^14−16^ Compared to power-law THz detectors (including those based on graphene^17−22^), the (homodyne) field detection of photomixers leads to a different scaling for the responsivity and noise equivalent power,^23^ having in principle a higher dynamic range and sensitivity (as the detected signal scales with , similar to heterodyne detection with a THz local oscillator).

**Figure 1:**
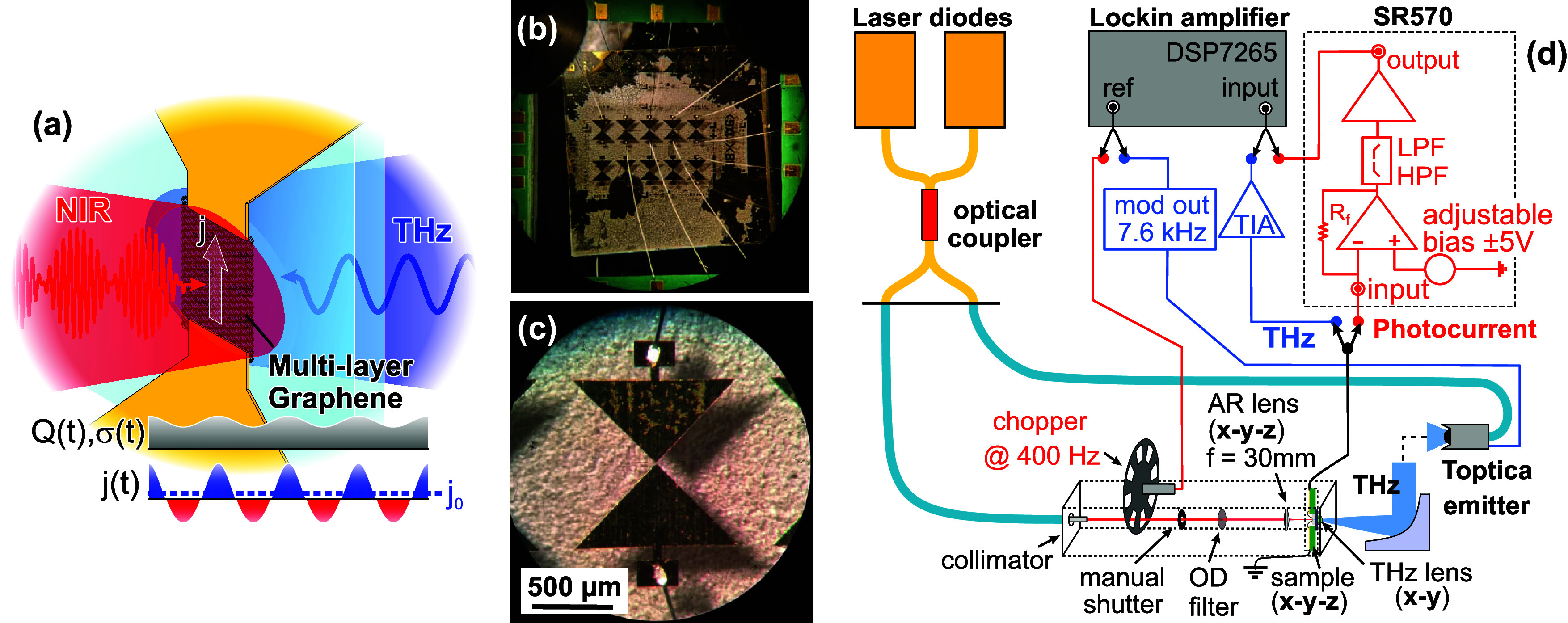
(a) Schematic of graphene photomixer for coherent THz detection via rectified photocurrent *j*_0_, due to mixing of conductivity modulation σ(*t*) (arising from the heat density *Q*(*t*) deposited by the two-color near-IR optical beat note) with the CW THz field in the gap (Si coupling lens for THz wave on back of substrate omitted for simplicity). (b) Micrograph of a chip with several packaged and bonded multilayer graphene photomixers and (c) magnified view of a single device where one can see the bow-tie antenna (as well as its shadow on the holder of the chip through the optically transparent sapphire substrate). (d) Schematic of the graphene-THz photomixer setup, including complementary circuitry for measurements of either the photocurrent (red, with variable bias and chopping of optical beam for lock-in detection) or THz signals (blue, both direct and photomixing signals with zero-bias and amplitude modulation of the THz emitter for lock-in detection).

We investigate both the photoconductivity and THz photomixing response of a multilayer graphene device and show that such coherent THz detection is possible. Here we chose a CVD-grown material composed of 6–8 layers, to achieve a greater degree of optical absorption compared to a single layer. The experiments are complemented with simulations which account near-quantitatively for the results and indicate avenues to improve the photomixing performance.

## Results

### Graphene Photomixer Devices and Measurement Apparatus

Figure 1b,c displays two micrographs of devices studied in this work. In Figure 1b, we show an array of packaged and bonded graphene photomixer devices fabricated on a sapphire substrate, with a magnified view of a single graphene photomixer in Figure 1c (see Materials and Methods for details of the fabrication). Not clearly discernible is the gap region (13.6 × 11 μm^2^) of the bow-tie antenna which contains 6–8 layers of commercial CVD-grown graphene as the photoconductive material (see Materials and Methods), while the graphene outside of the gap region was etched away in an oxygen plasma. The devices have no back gates, with the native graphene doping due to interaction between the substrate and other layers (see section on simulations below).

The photoresponse and the THz-photomixing properties of the devices were measured with a Toptica Terascan 1550 spectroscopy system, which usually employs a photoconductive InGaAs emitter and receiver, but where the standard receiver unit was replaced by our graphene device, as depicted in Figure 1d. As shown, we employ either of two external circuits to measure the DC photocurrent (red, with external bias voltage and optical chopping) and photomixing signals (blue, modulating the THz field via the emitter bias), respectively, the latter not requiring any external bias to measure the rectified photomixing signal, as per conventional THz photomixers.^14−16^ All experiments were performed at room temperature.

### Photocurrent Measurements

**Figure 2:**
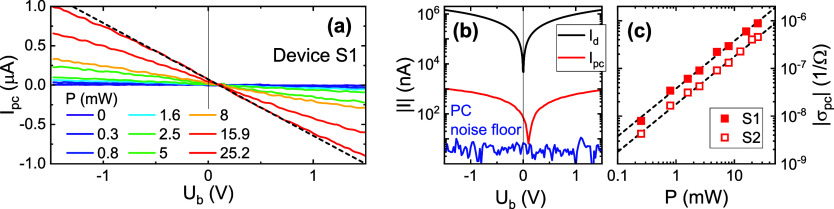
(a) Photocurrent measurements *I*_pc_ as a function of bias *U*_b_ for a series of incident optical power levels *P* of the two-color laser beam (device S1). (b) Comparison of photocurrent in panel a for the highest incident power (red curve) and dark current (black) (blue curve, photocurrent noise floor, lock-in integration time 100 ms). (c) Photoconductivity derived from the linear current–voltage regime of the data in panel a vs optical power *P* (including results for a second device, S2). Black dashed lines represent linear power dependence.

The dark current was measured vs bias voltage *U*_b_ and found to be perfectly linear vs bias, indicating essentially ideal ohmic resistance. The extracted dark resistance *R*_0_ of one of the graphene photomixers (device S1) was estimated to be *R*_0_ = 1.04 kΩ. Measurements of the photocurrent *I*_pc_ vs *U*_b_ for the same device S1 are shown in Figure 2a for a range of near-IR pump powers *P* (also shown for the highest *P* in Figure 2b for comparison with the dark current). For bias values |*U*_b_| < 0.6 V, the photocurrent is found to depend in a fairly linear manner on *U*_b_, while it shows a tendency toward saturation at higher bias. The corresponding photoconductivity σ_pc_ is obtained from the slope in the linear regime. Note that while we indeed measure the photoconductance, *G*_pc_ = 1/*R*_light_ – 1/*R*_dark_, here we do not distinguish this from the sheet photoconductivity σ_pc_, as with the square gap region the two quantities are essentially equal. The absolute value |σ_pc_| is presented in Figure 2c for two exemplary devices S1 and S2 (both prepared with the same material/procedure; we address the variation in performance below). For both, one observes a linear dependence vs incident power. The negative sign of σ_pc_ corresponds to photoinduced differential *resistivity*, as observed previously^1,4^ for graphene devices sufficiently away from the intrinsic doping regime (where one instead has positive photoconductivity). One also sees in Figure 2a that the sign of the photocurrent changes at *U*_b_ = 0.1 V instead of at zero bias. This shift is attributed to a residual asymmetry of the properties at the two gap contacts (e.g., due to electrode-induced doping gradients) which gives rise to an additional unipolar photocurrent component.^24,25^

### THz Detection

**Figure 3:**
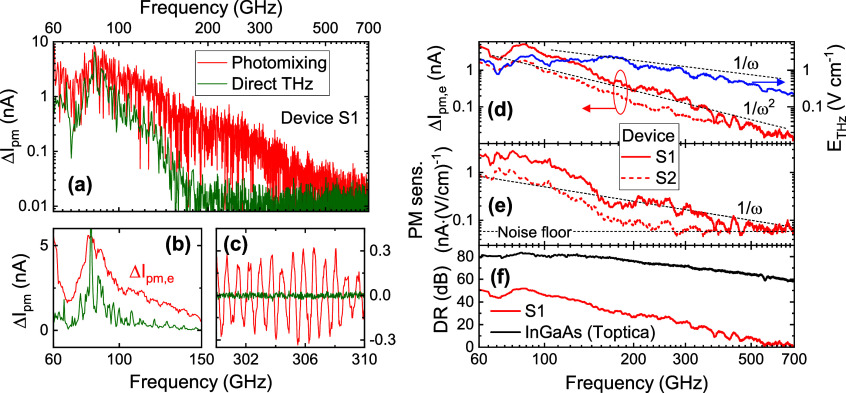
Experimental THz photomixer response vs THz-wave frequency (lock-in integration time: 100 ms). (a) Detected photomixer current Δ*I*_pm_ (red) after subtraction of direct-THz photocurrent (green, measured in the absence of the optical beat on the receiver) for device S1. Note that the fluctuations in the photomixer signal are dominated by the coherent interference oscillations, see panel b. (b, c) Magnified frequency ranges of signals (linear vertical scales; in panel b plotting the amplitude envelope Δ*I*_pm,e_). (d) Photomixer amplitude spectra (left scale) for both devices, S1 and S2 (solid and dashed curves, respectively), and estimated THz field amplitude in the photomixer gap (right scale) calculated using experimental THz power spectrum and antenna response (see text). Roll-off curves following 1/ω and 1/ω^2^ included for comparison. (e) Estimated photomixer sensitivity *R*_E_(ν) = Δ*I*_pm,e_(ν)/*E*_THz_ for each device, based on the results in panel d. (f) Comparison of the dynamic range (DR) achieved with the graphene photomixer (red curve, device S1) and a commercial InGaAs photomixer (Toptica spectrometer, black curve).

If one now operates the device without a bias voltage and illuminates the device with THz radiation (frequency ν) instead of the two-color near-IR laser radiation, one also obtains a photocurrent *I*_d_, termed “direct signal” in the following, as shown vs ν for device S1 in Figure 3a (green curve). This is measured by switching to the blue circuit in Figure 1d and modulating the THz wave via a square-wave bias on the emitter at 7.6 kHz for lock-in detection. The magnitude of the direct signal, unlike the photomixing signal to be discussed next, is proportional to the *power* of the incoming THz wave (see Supporting Information). The frequency dependence exhibits a strong roll-off toward high frequencies such that the photocurrent reaches the noise floor just below 200 GHz. This direct signal must result from a residual spatial asymmetry in the device and, being proportional to the THz power, likely arises from photovoltaic/photothermoelectric effects due to energy and charge-carrier diffusion at the (unequal) contact regions.^26^

Turning now to the photomixing detection, we illuminate the device simultaneously with both the THz wave and the near-IR beat note at the same frequency (still using the same circuitry and THz emitter modulation for lock-in detection, without chopping the near-IR beam). In this case, the measured photocurrent spectrum *I*(ν) changes to *I*(ν) = *I*_d_(ν) + Δ*I*_pm_(ν), i.e., with the additional signal Δ*I*_pm_(ν) shown by the red curve in Figure 3a (for both beams at the maximal available power), obtained by subtracting *I*_d_(ν) measured above from *I*(ν). For the employed lock-in integration time of 100 ms, the signal is above the noise floor from 60 to 700 GHz.

The magnified, low-frequency range in Figure 3b allows a clearer comparison of the direct and photomixer signals (in terms of the amplitude envelope Δ*I*_pm,e_ for the latter) at low frequencies. That the signal is of photomixing origin is demonstrated clearly by the oscillations in Δ*I*_pm_ vs frequency (which varies with the relative phase φ = ω·δ*t*, δ*t* being the delay between THz and optical beat note at the detector); a magnified range is shown in Figure 3c (where the direct signal has already dropped to the noise floor). Note that the slower modulation on the envelope of the interference signal is due to reflections in the THz and optical beam paths (as also seen using a commercial photomixer for detection). In Figure 3d, we plot Δ*I*_pm,e_(ν) extracted from the Δ*I*_pm_(ν) in Figure 3a, as well as the corresponding data for the second device S2. As per the photocurrent in Figure 2c, device S2 shows a lower photomixing response. While one might attribute this simply to a different number of layers (i.e., ∼8 layers for S1 and ∼6 layers for S2), the situation seems more complex, given that the ratios (S1/S2) of the dark conductivity (1.4:1), photoconductivity (1.9:1), and photomixing response (2.9:1) are different. Hence we speculate that variations in the layer stacking/doping and possibly contact resistances also lead to such differences between samples. In Figure 3d, one sees the spectra roll-off with increasing frequency, with a rate close to 1/ω^2^ above 200 GHz, which is partly due to the roll-off of the THz emitter field spectrum *E*_THz_(ν) (which also has a dip in the emitter field strength near 70 GHz). This roll-off affects both the direct-THz (∝*E*_THz_^2^) and photomixer (∝*E*_THz_) spectra in Figure 3a. In order to calculate the inherent photomixing sensitivity *R*_E_(ν) = Δ*I*_pm,e_(ν)/*E*_THz_(ν) of the device (*E*_THz_ being the amplitude of the THz radiation field in the photomixer gap), we combined both the experimentally measured THz power spectrum and simulations of the THz radiation coupling efficiency to estimate *E*_THz_. These simulations take into consideration the coupling efficiency of the Si substrate lens and antenna response (see Materials and Methods). The resultant spectrum *E*_THz_(ν) is shown in Figure 3d (blue curve, right scale), which exhibits roughly a 1/ω roll-off due to the frequency characteristics of the photoconductive-emitter source. While the estimate of *R*_E_(ν) may be prone to systematic errors due to the involved procedure to estimate the effective THz field in the gap, we present the nominal results here as they can be compared directly with the simulation results below.

We then arrive at the photomixer sensitivity *R*_E_(ν) shown in Figure 3e for the two devices. The highest sensitivity is reached at 79 GHz, with a photodetector sensitivity of 2.2 nA/(V/cm). Having compensated for the roll-off of the THz field spectrum, one sees that *R*_E_ roughly follows a 1/ω-dependence above 200 GHz. As we will discuss on the basis of simulations below, this dependence is a consequence of the finite energy relaxation time τ_c_ of the charge carriers of graphene. Note that the enhanced photomixer contribution below 200 GHz is likely to arise from the wiring of the bow-tie antenna^27^ and not the bow-tie antenna coupling efficiency (which simulations show is relatively flat over the full spectral range here, i.e., only varying by 1.5 dB, see Materials and Methods).

In Figure 3f, we plot the dynamic-range (DR) spectrum calculated from the photomixing signal spectrum in Figure 3d for device S1 (red curve), calculated as DR = 20 log_10_(Δ*I*_pm,e_(ν)/Δ*I*_pm0_) (i.e., referenced to THz power), where Δ*I*_pm0_ is the noise level calculated from the standard deviation of the signal in the absence of THz/optical power on the device. The peak DR value is 52 dB measured at 86 GHz. To put this performance into perspective, we also plot the DR spectrum for detection with the InGaAs photomixer of the Toptica measurement unit (black curve). It exhibits a 32 dB higher current response at 86 GHz, growing to 50 dB at 200 GHz due to the roll-off of the graphene sensitivity. These and additional measurements with other graphene photomixers show that the graphene devices achieve an appreciable THz photomixing response, but still with a sensitivity and DR substantially lower than that of the InGaAs device. However, this comparison has to be taken in context, as one puts a highly optimized photomixer against an unoptimized one; e.g., the Toptica device uses optical-fiber coupling of the two-color near-IR radiation instead of the less-efficient free-space coupling in the case of the graphene photomixer. In the next section, we simulate the performance of the graphene device in order to understand the experimental results. We then identify substantial potential for improved performance, in particular, by optimization of the doping level.

### Simulated Photocurrent and Photomixing Response

In order to theoretically describe and quantify the photomixing process, we apply a modified version of the time-dependent conductivity model described in refs (9 and 10), where here the modulation in the carrier heating from the two-color optical pump yields a periodic change in the conductivity which is then driven by (i.e., mixes with) the THz field to yield the detected DC current signal. A detailed description of the band structure and doping levels for such a multilayer graphene system is nontrivial, as the adjacent layers may form a composite gapped band structure,^28^ while even for weakly interacting layers one has to consider how substrate- and contact-induced doping will distribute across the layers, including the effects of screening. For simplicity, we model the multilayer graphene sample as a set of parallel layers (sheet conductances), each represented by the linear dispersion of single-layer graphene, with the same nominal doping and scattering rates.

It is well-established that for moderate excitation levels, single-layer graphene exhibits positive photoconductivity for low doping levels, which crosses over to negative photoconductivity (i.e., photoresistance) at higher doping, the latter as seen in our graphene devices (Figure 2a). While this was first attributed to contributions from both photovoltaic effects (by optically excited electron–hole (e–h) pairs) and bolometric effects (by a change in carrier distribution due to heating),^1^ respectively, it was shown subsequently^4^ that both regimes can be attributed to a bolometric response when accounting for the change in both the carrier temperature *T*_el_ and chemical potential *E*_F_ of the hot carriers. Underlying this line of argument is that the charge carriers rapidly relax after photoexcitation on a time scale ≲100 fs and remain close to single hot Fermi–Dirac distribution *f*(*E*, *T*_el_) (across both valence and conduction bands) due to rapid electron–electron (e–e) scattering, with a heat density *Q* given by the absorbed optical intensity.^4^ It is hence assumed in the following that the charge carriers are thermalized and that at any time one can assign a temperature *T*_el_ to the carrier ensemble.

**Figure 4:**
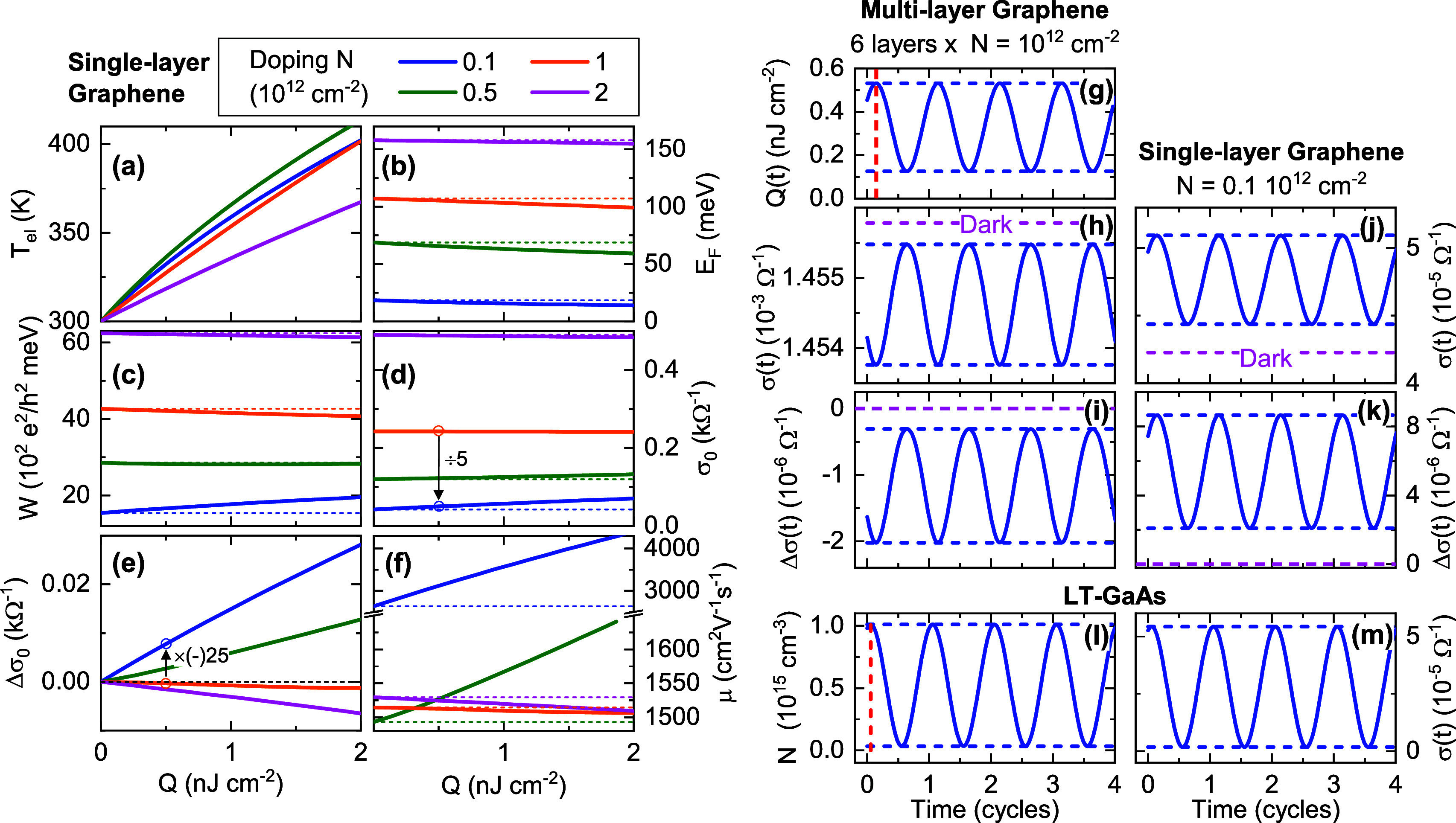
(a–f) Dependence of parameters for the carrier distribution and conductivity on heat density *Q* for a set of doping levels *N* in a single graphene layer, as used for the photoconductivity/photomixer simulations. (a) Temperature *T*_el_, (b) chemical potential *E*_F_, (c) Drude spectral weight *W*, (d) DC conductivity (2D) σ_0_, (e) differential conductivity Δσ_0_ (i.e., photoconductivity) in panel d, and (f) effective mobility μ (based on doping density *N*). Dashed horizontal lines for respective values for *Q* = 0. Arrows and multiplication factors in panels d and e in going from *N* = 10^12^ cm^–2^ to *N* = 0.1 × 10^12^ cm^–2^ (for *Q* = 0.5 nJ cm^–2^); see Discussion. (g–m) Simulated time dependencies for the highest experimental power in Figure 2 and a laser beat frequency of Δν = 200 GHz. (g) Optically deposited heat density (average per layer), (h) total and (i) differential sheet conductivity of the modeled 6-layer graphene device. (j, k) Corresponding simulated results as in panels h and i, respectively, but for single-layer graphene with near-intrinsic doping. (l, m) Complementary results for a LT-GaAs photomixer with the same optical pump intensity and beat frequency (see text): (l) volume density of excited e–h pairs in the surface region and (m) time-dependent sheet conductivity. Vertical dashed lines in panels g and l to demonstrate the phase shift relative to the optical beat signal maxima at *t* = 0.

The expression for the conductivity of each layer, based on the generalized Drude model with a hot thermal carrier distribution is given in the Materials and Methods section (eq 1), with *T*_el_ and *E*_F_ determined by *Q* with fixed doping concentration (net carrier density) *N*. For the calculation of the sheet (2D) electric conductivity σ(ω) and the mobility μ(ω), one requires the carrier-energy-dependent momentum scattering rate, Γ(*E*). Here, we include acoustic- and optical-phonon scattering, in addition to charged-impurity scattering, Γ_i_(*E*),^9,29^ the latter to represent both impurities in the substrate as well as interlayer interactions (see Materials and Methods). Results from this treatment are shown in Figure 4 for *T* = 300 K vs *Q* for a set of doping levels *N* (note that the experimental optical intensity levels above correspond to a maximum of *Q* < 0.5 nJ cm^–2^, see below). In Figure 4a,b, one observes (i) the dependence of *T*_el_(*Q*), which first becomes steeper and then less steep for small *N*, and hence is nonmonotonic in *N*, due to a local maximum in the electronic heat capacity *C*_el_(*N*) at finite *T*_el_,^5^ and (ii) the reduction in chemical potential *E*_F_(*Q*) with increasing *Q*, as required to conserve the total charge density.

Turning now to the sheet conductivity σ = Re{σ̃}, we first plot the spectral weight *W* = ∫_0_^∞^ σ(ω) dω in Figure 4c), which one can show depends only on the Fermi–Dirac distribution *f*(*E*, *T*_el_) of the carriers (and not Γ(*E*)), and hence allows more universal initial statements about the changes of the conductivity vs parameters such as *Q*.^4^ In Figure 4c, one observes that the slope of *W*(*Q*), which is a measure for the photoconductivity, changes from positive to negative at *N* ∼ 0.5 × 10^12^ cm^–2^. This behavior results from the detailed dependence of ∂_E_*f* for the carrier distribution, i.e., the density of neighboring vacant states for intraband scattering. At much higher *Q* (outside of the plotted range and corresponding to *T*_el_ ≳ 1000 K), *W*(*Q*) becomes an increasing function of *Q* for all *N*.^4^

The absolute DC conductivity σ_0_ = σ(ω = 0) and DC photoconductivity (given by the differential Δσ_0_ = σ_0_(*Q*) – σ_0_(*Q* = 0)), are shown in Figure 4d,e, respectively. Here, one also sees the crossover behavior vs *N*, albeit at a slightly larger *N* > 1 × 10^12^ cm^–2^. One also finds that the photoconductivity has a larger gradient dΔσ_0_/d*Q* for small *N*, as per *W*(*Q*). A crossover is also seen in the slope of the mobility μ, which is included for completeness in Figure 4f. (Note that the nontrivial trend of μ(*Q* = 0, *N*) results from the fact that thermal excitation introduces more band carriers at *T* = 300 K than the nominal net doping *N* for low *N*, while at higher *N*, the energy dependence of the impurity scattering rate ∝|*E*|^–1^ still dominates over the onset of phonon scattering).

As mentioned above, we represent the multilayer device as composed of 6 identical graphene layers. A doping level of *N* = 1 × 10^12^ cm^–2^ in each layer was chosen, which in combination with the assumed impurity scattering rate and cooling rate yields magnitudes of the DC resistance and photoconductivity close to those in the experiment. Using these results, we can calculate the time-dependent photoconductivity response to the THz wave due to heating by the absorbed optical pump intensity. Based on the experimental beam diameter and power, we calculate the average intensity *I*_1,2_ in the electrode gap for each component of the two-color optical pump (amounting to *I*_1,2_ = 74 W mm^–2^ for the maximum total pump power *P* = 25.2 mW in Figure 2a). The time-dependent heat density *Q*_*n*_(*t*) in the *n*th layer is calculated by solving eq 2 (Materials and Methods) for the absorption of optical intensity and cooling due to energy relaxation by electron–phonon (e–ph) scattering, for which we assume a time constant of τ_c_ = 1 ps, based on literature reports.^4,5,30^ Here we assume that the absorbed optical energy is initially preserved in the electronic system during rapid elastic e–e scattering, with e–ph scattering contributing only to the subsequent cooling (energy relaxation), which is a reasonable approximation for the relatively weakly doped graphene layers here.^31^ As the lattice heat capacity is 2–4 orders larger than the electronic one,^5^ we treat the lattice as an ideal reservoir and neglect any further cooling bottlenecks (with the substrate ultimately extracting the lattice heat with only a small increase in lattice temperature^5^).

The resultant time-dependent heat density *Q*(*t*) and conductivity σ(*t*) (again, for the maximum experimental total power *P* = 25.2 mW in Figure 2a) is shown in Figure 4g–i for a laser beat frequency of Δν = 200 GHz. Here one sees that the cooling is sufficiently fast that the heat density modulation depth is high (1 – *Q*_min_/*Q*_max_ = 0.76), resulting in a peak–valley variation in the total multilayer conductivity of Δσ_pp_ = 1.7 × 10^–6^ Ω^–1^ (Figure 4i). The relative conductivity modulation amounts to Δσ_pp_/σ = 1.2 × 10^–3^. For comparison, in Figure 4j,k, we plot the corresponding results for a *single* graphene layer, but for a ten times lower doping density *N* = 0.1 × 10^12^ cm^–2^ closer to intrinsic. As expected from Figure 4e, the polarity now corresponds to positive photoconductivity, and Δσ_pp_ = 6.6 × 10^–6^ Ω^–1^ is a factor 3.9 times higher, despite only having a single layer (see Discussion below).

To put the magnitude of this conductivity modulation into perspective, we performed complementary simulations of photoexcited low-temperature-grown LT-GaAs, where the photoconductivity modulation arises due to interband e–h generation and subsequent carrier trapping, using the same optical intensity and laser beat frequency (assuming an excitation wavelength close to the bandgap). We assumed a momentum relaxation rate of 1/τ, with τ = 160 fs and a carrier trapping time (mobility lifetime) of τ_c_ = 300 fs, see Materials and Methods. The results are shown in Figure 4l,m. One sees that the surface excitation densities for such a CW photomixer are only moderate (reaching values of *N*_e,h_ ∼ 10^15^ cm^–3^, significantly lower than that reached with femtosecond pulsed excitation^32^), and when one integrates the total sheet conductivity, this amounts to Δσ_pp_ ∼ 50 × 10^–6^ Ω^–1^ (Figure 4m), i.e., a factor 30 larger than we calculate for our present multilayer graphene device, but only a factor ∼10 larger than that predicted for a single-layer device tuned to near-intrinsic doping. Hence, given that only 2.3% of the optical power is absorbed in a single graphene layer (compared to complete absorption in the LT-GaAs surface layer), one sees that a comparable sheet photoconductivity per unit absorbed power is predicted.

**Figure 5:**
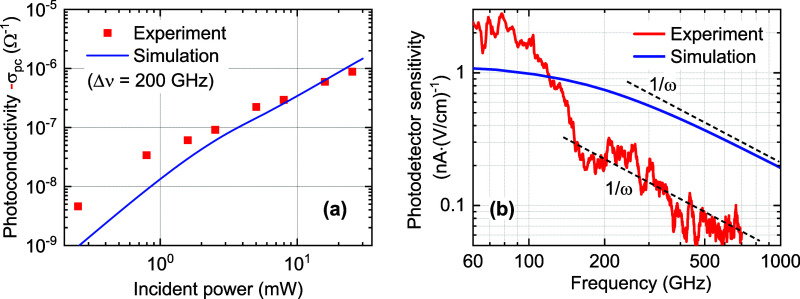
(a) Simulated and experimental photoconductivity vs optical power for the multilayer graphene device with experimental conditions as in Figure 2. (b) Simulated and experimental (Figure 3e) photomixer field sensitivity vs THz frequency. Lines corresponding to a first-order response roll-off (∝ 1/ω) added as visual guides.

On the basis of these results, we can calculate the predicted DC photoconductivity of the graphene device, by taking the cycle-average value of σ_pc_ = ⟨Δσ(*t*)⟩ (as we employ a nearly square-shaped device gap, the sheet conductivity also corresponds to the device conductance). This is shown in Figure 5a along with the experimental results from Figure 2c for the same conditions. Note that our choice of the doping level was guided by the magnitude of the experimental photocurrent, and hence the agreement seen is to be expected, although the result is also based on choosing reasonable values for the impurity scattering rate and cooling rate and reproducing the dark DC resistance of the device. Moreover, although there is a deviation between experiment and simulation at low power, the saturation behavior is present in both.

Finally, we calculate the predicted THz photomixer sensitivity *R*_E_(ν) of the device on the basis of the simulation results above, for comparison with the experimental results in Figure 3e. If one considers a homogeneous THz electric field coupled into the electrode gap (replacing the DC bias used to measure the DC photocurrent above), *E*(*t*) = *E*_0_ cos(ω*t*), then the current density is given by a convolution of the driving field *E*(*t*) with a Drude response whose parameters are modulated as per *Q*(*t*) due to the two-color optical excitation. In general, this would allow for mixing terms with the modulation of both the plasma frequency and Drude scattering rate of the electron–hole gas. However, given the short momentum scattering time (≪100 fs), the leading term can be expressed in terms of a time-dependent (parametric) conductivity, i.e., the quantity σ(*t*) presented above (Figure 4h). One can then show that the rectified (DC) current density amplitude is given by  (achieved for the optimal relative phase between the THz and optical beat by scanning their relative delay at the detector, see Materials and Methods), with a resulting device current *I* = *w*·*j* (with the electrode width *w* = 13.6 μm). The simulated sensitivity spectrum is shown in Figure 5b, as well as the experimental one from Figure 3e. Although the simulation parameters were chosen to correspond to the experimental photocurrent in Figure 5a, no further extensions/modifications were employed to predict the THz sensitivity. In this sense, the agreement in Figure 5b provides good independent support for the foregoing model, despite its simplifications. Good agreement is reached regarding the roll-off of the signal. The simulated sensitivity spectrum possesses a first-order (∝1/ω) roll-off with a 3 dB bandwidth of ∼300 GHz, due to the electron cooling model used with τ_c_ = 1 ps. The experimental spectrum shows the predicted 1/ω roll-off at high frequencies. The higher-than-predicted (and frequency-modulated) sensitivity below ∼150 GHz is attributed mainly to the antenna effect of the wiring of the detector, compare discussion of the photomixer sensitivity *R*_E_(ν) as shown in Figure 3e.

## Discussion

The predictions here indicate a path to significantly enhance the graphene photomixing performance. Before addressing this potential, we put this performance into perspective with regard to conventional semiconductor-based photomixers. On the most basic level, the conductive nature of the graphene photomixer needs to be discussed. With photomixers, one commonly strives for high dark resistance of the devices, such that the optically induced conductance modulation is as close to 100% as possible (compare Figure 4l,m for LT-GaAs) and the noise floor is as low as possible.^33^ The noise current scales with , with σ(*t*), as above, being the time-dependent conductivity under illumination.^33^ Comparing the values of ⟨σ(*t*)⟩ for the graphene and LT-GaAs photomixers of the simulations for 200 GHz in Figure 4h and Figure 4m, respectively, the average bright conductivity (1.46 × 10^–3^ Ω^–1^) of the graphene device is about a factor 50 larger than that of the LT-GaAs device (2.5 × 10^–5^ Ω^–1^); the noise current is hence expected to be about 7–8 times larger. How does this play out in the device performance? Here, the relevant quantity is the noise-equivalent power (NEP). One derives for photomixers (which are field and not power detectors) that the NEP (with units W/Hz) has the following dependence:^33^ NEP ∝ ⟨σ(t)⟩/(Δσ_pp_)^2^, with Δσ_pp_ being the modulation depth of the conductivity (see above). The calculated value of Δσ_pp_ of the graphene device is about 50 times smaller than the corresponding value for the LT-GaAs photomixer (compare Figure 4i vs Figure 4m). One then finds that NEP_graphene_/NEP_LT-GaAs_ ≈ 1.3 × 10^5^ = 51 dB. This is indeed close to the difference in dynamic range which one deduces at 200 GHz from Figure 3f with regard to the standard InGaAs photomixer of the Toptica spectroscopy system, whose performance should be similar to that of the modeled LT-GaAs device. (Note that the optimal absolute NEP of the graphene device S1 was determined to be 162 pW/Hz at 78 GHz, with a corresponding responsivity of ).

By how much can the performance as expressed by the NEP be improved in the 200 GHz range and below? The key direction here would be to develop a photomixer with a single (hBN-encapsulated) layer of graphene and with control of the density of the charge carriers by a (back)gate voltage to approach the intrinsic regime, which yields simultaneous improvements in both the predicted photoconductivity and average conductivity, as indicated in Figure 4d,e in going from *N* = 10^12^ cm^–2^ to *N* = 0.1 × 10^12^ cm^–2^ (at a representative heat density of *Q* = 0.5 nJ cm^–2^). Here we predict that Δσ_pp_ can increase by a factor of ∼25 per layer, i.e., still a factor ∼4 for the sensitivity of a single-layer device (compared to the 6-layer device here). In addition, the dark conductivity (and hence the scale for ⟨σ(*t*)⟩) reduces by a factor 5 per layer or a factor 30 in going to a single layer (here the reduction of layers being beneficial), which will decrease the noise level. Combined, this translates into a predicted NEP improvement by a factor of 520 or 27 dB for a near-intrinsic single-layer device. Moreover, designs to exploit plasmonic effects to enhance the near-IR absorption should offer further performance enhancement.^2,34^ If, as in ref (2), an absorption of 75% could be reached (while maintaining the ultrafast energy relaxation of the charge carriers), this would translate into a further reduction of the NEP by as much as a factor of about 33^2^ = 1000 (or 30 dB). Additional improvement of the response is to be expected if one enhances the strength of the THz electric field in the gap of the antenna, e.g., using interdigitated electrodes,^35^ as is the standard of most modern photomixers, or employing waveguide geometries for the THz wave.^36,37^ Also, professional packaging of the device including optical-fiber coupling of the optical radiation will improve the performance. There is hence much room for the development of highly sensitive graphene photomixers, such that our current NEP value could reach well into the fW/Hz range, as per more mature, optimized THz photomixers.^14^

It has to be noted, however, that THz detection with a graphene photomixer will have a faster frequency roll-off than the detection with (optimally mobility-lifetime-engineered) semiconductor-based devices because of the longer relaxation time constant τ_c_ (note that τ_c_ further increases at lower temperature^5^). The graphene photomixer is hence mainly of interest for applications with sub-500 GHz radiation. There it can play out its advantages, of which the most important are that it is easy to fabricate (especially not requiring the expensive growth of dedicated materials by atomic-layer growth techniques such as molecular beam epitaxy) and that it can be operated in combination with laser radiation over a broad wavelength range (from mid-IR to UV), which is also used for the generation of THz waves by photomixing.

As a final remark, we note that the ability here to enhance the photomixing response by approaching the intrinsic regime contrasts with the case of the self-driven THz nonlinearity for harmonic generation,^9^ where the optimal doping is well removed from the intrinsic regime.^10^ This is due to the fact that our heating modulation is driven independently by interband optical excitation, as opposed to intraband THz excitation, the latter depending not only on the sensitivity to heating but also on the absolute magnitude of the intraband conductivity which dictates the heat absorption and drops significantly as one approaches the intrinsic regime (Figure 4c).

## Conclusion

We have explored a photomixing mechanism for the detection of continuous-wave THz radiation. Unlike classical semiconductor-based photomixers which rely on interband generation of mobile charge carriers, we employ the hot-carrier bolometric effect in graphene. Its ultrafast response gives rise to a “relaxational” nonlinearity which is used for the rectification of the THz radiation. This mechanism is related to mixing in conventional hot-electron bolometers (HEBs);^38^ while these require cryogenic cooling, the photomixing with graphene works at room temperature. Good agreement is found between the experimental data and simulations based on an energy-relaxation model of the charge carriers in graphene. Variation of model parameters has allowed us to make suggestions for the improvement of the photomixer detector. From a practical point of view, such devices have the advantages of their ease of fabrication and of their ability to be operated with (local-oscillator) laser radiation at arbitrary wavelengths.

## Materials and Methods

### Detector Fabrication

The devices were fabricated on sapphire substrates (thickness 300 μm), which were first cleaned in an acetone and isopropanol bath. Upon this we deposited the multilayer graphene sheet, which is composed of 6–8 layers of CVD-grown graphene, obtained commercially from ACS Material LLC (trade name: Trivial Transfer Graphene, provided already transferred from the Cu-foil to a PMMA/polymer sandwich), followed by photolithographic definition of the bow-tie antenna (300 nm thick Au film on a 2 nm thick Ti adhesion layer, antenna arm length = 493 μm, bow-tie flare angle = 90.8°, gap = 13.6 × 11 μm^2^. As the final step, the graphene was etched away in an oxygen plasma treatment everywhere except in the antenna gap. A photoresist layer protected the gap region during plasma treatment and was removed after the etch step.

### Photocurrent and THz Measurement Setup

All optical measurements were performed with a CW THz spectrometer, Terascan 1550 (vendor: Toptica Photonics AG, Gräfelfing/München). It uses two diode lasers operating at wavelengths in the telecom wavelength range (λ = 1550 nm). The fiber-coupled InGaAs photomixer serving as THz detector in the system was replaced by an optical bench for free-space illumination of our graphene photomixer device as illustrated in Figure 1d. The bench (a four-steel-rod cage) contained a collimation lens (AR-coated for 1550 nm, producing a collimated beam with a 1/*e*^2^ diameter of 3.6 mm, a mechanical light chopper, an aperture, filters for beam attenuation and a AR-coated lens (*f* = 30 mm) to focus the laser radiation into the gap of the photomixer antenna. The lens was mounted on a *xyz*-translation stage for beam centering and optical prealignment. This is decisive for subsequent optimization of the photomixer signal, without affecting the prealignment of the photocurrent. The detector (held at room temperature and exposed to air, no purging of the beam path to remove water vapor) was mounted on a second *xyz*-translation stage (a 3-axis, high-precision flexure stage, Thorlabs NanoMax) for the alignment of the laser beam’s focus into the antenna gap.

For the photocurrent measurements, the laser beam was chopped at 400 Hz by the mechanical chopper. The maximum beam power before the chopper was determined with an integrating sphere photodiode power sensor (Thorlabs Inc., S145C). The power was then varied by inserting filters with different optical density (OD). The photocurrent, *I*_pc_, was measured with one antenna leaf grounded and the other connected to the internal bias source (which provided the bias voltage *U*_b_) of a low-noise current preamplifier (model SR570 from Stanford Research Systems). The optimization of the alignment of the optical beam was performed at a fixed bias of 0.6 V. The current amplifier was operated in high-pass (HP) mode (filter corner frequency = 100 Hz, filter slope = 6 dB, sensitivity = 500 μA/V, gain mode = low noise (LN)). Its output signal was fed to a lock-in amplifier (Ametek DSP 7265). The lock-in integration time constant was set to integration time 100 ms. The peak-to-peak photocurrent was determined as , with *I*_lock-in_ being the current reading of the lock-in amplifier.

For the THz sensitivity characterization, a hyperhemispherical Si-substrate lens with a diameter of 12 mm was mounted in a freely positionable way (not glued) against the back-side of the detector.^39^ The lens was mounted on another *xy*-stage used for the alignment of the lens in the THz beam. Special care was taken to center the THz beam on the gap region of the antenna prior to the fine-tuning of the substrate lens (in order to ensure good focusing at all THz frequencies). The rectified current amplitude due to the THz field was measured with a lock-in amplifier at the modulation frequency (7.6 kHz) of the InGaAs photomixer emitter bias (Toptica) with an integration time of 100 ms. The current amplitude was determined from .^39^

### Estimation of THz Field Amplitude in Photomixer Gap

The amplitude of the THz electric field in the antenna gap is calculated from the voltage *U*_THz_ of the THz signal across the gap as *E*_THz_ = *U*_THz_/*d*_gap_, the gap in the bow-tie antenna having the length *d*_gap_ = 13.6 μm. *U*_THz_ is determined from the power of the incident THz radiation via . Here, *R*_0_ = 1040 Ω is the ohmic resistance of the graphene stripe in the gap, and *P*_THz_ is the power of the THz beam delivered to the gap. *P*_THz_ is calculated via^39,40^
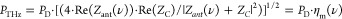
*Z*_ant_(ν) and *Z*_C_ denote the impedances of the antenna and the graphene channel, respectively.^39^ We assume the latter to be real-valued and constant in frequency, *Z*_C_ = *R*_0_. The frequency-dependent antenna impedance *Z*_ant_(ν) is obtained from EM simulations (Keysight Advanced Design System (ADS)), see Supporting Information. *P*_D_ is the fraction of the total free-space beam power *P*_THz_^op^ which is collected by the antenna and can be determined from *P*_D_ = (1/2)·η_op_·η_gauss_·η_ant_(ν)·*P*_THz_^op^,^39,41^ such that the total power delivered to the multilayer graphene is obtained from *P*_THz_ = η_tot_(ν)·*P*_THz_^op^, where the total coupling efficiency, given by η_tot_(ν) = (1/2)·η_op_·η_gauss_·η_ant_(ν)·η_m_(ν), accounts for all losses toward the antenna gap. The free-space THz power *P*_THz_^op^ from the Toptica emitter, incident at the air-to-silicon-lens interface of the detector, was measured with a calibrated Golay cell across the range 60–700 GHz, with the following values: 29.7 μW (60 GHz); 5.5 μW (70 GHz, local dip); 29.2 μW (80 GHz); 45.9 μW (90 GHz, spectral maximum); 5.9 μW (300 GHz); 0.35 μW (700 GHz). The frequency-dependent antenna efficiency η_ant_(ν) of the bow-tie antenna is also derived from EM simulations (ADS). η_op_ = 0.7 is the optical power loss factor accounting for reflection losses at the air-to-silicon-lens interface,^39^ η_gauss_ = 0.9 denotes the Gaussian-beam coupling efficiency,^39,42^ also known as Gaussicity, and the factor of 1/2 accounts for residual scattering of incident power in a receiving antenna element.^40,43^ Example values at the frequencies 100, 300, and 700 GHz for the total coupling efficiency are η_tot_(ν) of 2.1, 2.3, and 2.4%, respectively.

### Theoretical Approach

The frequency-dependent, complex conductivity of each graphene layer is calculated via the generalized Drude response for each respective Fermi–Dirac carrier distribution:^9^
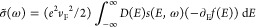
1where *D*(*E*) = 2|*E*|/π(ℏ*v*_F_)^2^ is the 2D density of states and *s*(*E*, ω) = (Γ(*E*) + *i*ω)^−1^ represents the differential Drude response at each energy *E*, and Γ is the energy-dependent momentum scattering rate. The Fermi–Dirac distribution *f*(*E*) = [1 + e^β(*E*–*E*_F_)^]^−1^ with β = (*k*_B_*T*_el_)^−1^ is calculated by imposing the conditions for the total energy *U* = *U*_0_ + *Q* and constant doping density *N*(4,44) to determine *T*_el_(*Q*) and *E*_F_(*Q*), where *Q* is the instantaneous heat density absorbed in each respective layer. For the impurity scattering rate, we take Γ_i_ = (γ(*E*)|*E*|)^−1^, where γ(*E*) is a slowly varying function of *E*(29) and is found to vary for different graphene samples. Here we tuned the value of γ to yield the internal dark resistance of our experimental multilayer device (*R*_0_ – 2*R*_c_ = 635 Ω, assuming a series contact resistance *R*_c_ of 200 Ω at each electrode), corresponding to γ(*E* = 0) = 0.12 fs meV^–1^, which results in a magnitude for Γ_i_(*E*) intermediate between the values found in refs (9 and 29).

For calculating the time-dependent heat density *Q*_*n*_(*t*) in the *n*th graphene layer, we employ a simple single-component cooling rate with τ_c_ = 1 ps and solve the heat equation:
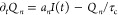
2where *a*_*n*_ is the absorption fraction in the *n*th layer, including the weak depletion of the light for each subsequent layer approaching the substrate (*a*_6_ = 2.29% for the uppermost layer). For our CVD-grown material, previous reports show that the interband optical absorption (for normally incidence light) corresponds closely to that of noninteracting single layers.^45,46^ As we assume the cooling rate is independent of *Q*, one can solve for *Q*(*t*) analytically with the incident two-color optical pump,
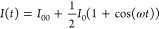
where ω = 2πΔν and Δν = ν_2_ – ν_1_ is the frequency difference between the two lasers. The expression for the rectified photomixer current density given in the main text can be derived as follows (similar to the treatment in ref (9)): In general, the time-dependent current density is given by *j*(*t*) = *∫σ*(*t*, *t*′)*E*_THz_(*t*′) d*t*′, or defining σ(*t*, ω) = *∫*e^*i*ω(*t*′^^–*t*)^σ(*t*, *t*′) d*t*′, one has
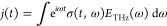
3For a Drude response with a fast scattering rate (fulfilling *ωτ* ≪ 1, as is the case for our spectral range), one can approximate the conductivity as the DC limit with a parametric time dependence, σ(*t*, ω) → σ(*t*, ω = 0) ≡ σ(*t*) and hence eq 3 simplifies to *j*(*t*) = σ(*t*)·*E*_THz_(*t*). For the THz field, we have 
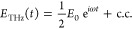
and to first-order both *Q*(*t*) (solution to eq 2) and σ(*t*) have the same time-dependence, i.e., for the latter we can write 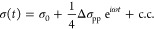
where Δσ_pp_ = |Δσ_pp_| e^*i*φ^. Then the ansatz 
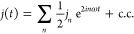
yields  for the rectified (DC) current density.

For the comparative calculations for a conventional semiconductor (LT-GaAs) photomixer, we take a carrier trapping time of τ_c_ = 300 fs, a Drude scattering time of τ = 160 fs, and the literature effective masses^47^ (yielding a reduced plasma mass of *m*_eff_ = (1/*m*_e_ + 1/*m*_h_)^–1^ = 0.056*m*_e0_). We assume carrier heating to be negligible and also that the bulk temperature remains close to room temperature (although there will be some lattice heating because a considerable part of the photon energy will be transferred to lattice vibrations by nonradiative energy relaxation processes).

## Data Availability

Both the experimental and simulation data and details concerning calibration/analyses can be obtained from the authors upon reasonable request.

## Supporting Information Available

Simulated photomixer antenna impedance spectrum, direct THz signal vs THz power, and measured THz power spectrum (PDF)
