# Supplementary material for: Coherent Terahertz Detection via Ultrafast Dynamics of Hot Dirac Fermions in Graphene
Source: ACS Nano. 2024 Feb 1;18(6):4765–74. doi: 10.1021/acsnano.3c08731 (PMC10868588; doi:10.1021/acsnano.3c08731)
Supplement: Supplementary file 1 — nn3c08731_si_001.pdf [file nn3c08731_si_001.pdf]

Supporting Information to:
Coherent terahertz detection via ultrafast dynamics of hot Dirac fermions in graphene

Mark D. Thomson,[1] Florian Ludwig,[1] Jakob Holstein,[1] Reiam Al-Mudhafar,[1,*] Shihab Al-Daffaie,[2] and Hartmut G. Roskos[1]

[1]*Physikalisches Institut, Johann Wolfgang Goethe-Universität, 60438 Frankfurt am Main, Germany*
[2]*Department of Electrical Engineering and Center for Terahertz Science and Technology, Eindhoven University of Technology, 5612 AE Eindhoven, Netherlands*

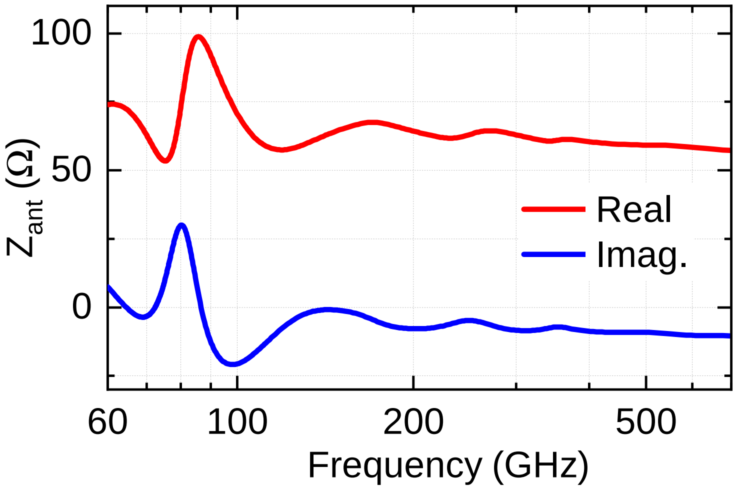

FIG. S1. Simulated impedance for bow-tie antenna (on sapphire substrate) as used for graphene photomixers in main paper (using the electromagnetism simulation software: Keysight Advanced Design System, ADS).

* on leave from the Institute of Laser, University of Baghdad, 79CH+WM Baghdad, Iraq

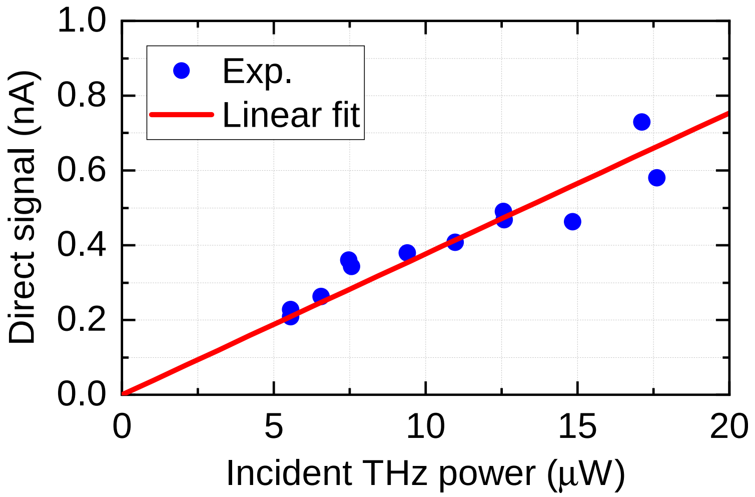

FIG. S2. Measured direct-signal current (*i.e.*, with no optical beat note for photomixing) vs. incident terahertz (THz) power (device S1) for a frequency ν = 70±3 GHz and linear fit. See Fig. 3(a,b) in main paper.

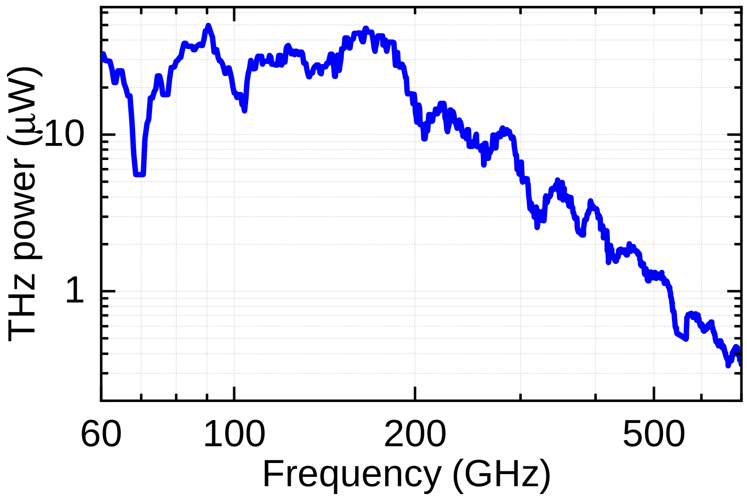

FIG. S3. Measured THz power spectrum of the Toptica TeraScan 1500 emitter (incident on photomixer detector), measured with a calibrated Golay cell. Used to calculate photomixer field responsivity and noise-equivalent-power (NEP) values in main paper.
